# Prevalence of chronic obstructive pulmonary disease in patients with catheter-diagnosed coronary artery disease

**DOI:** 10.4103/1817-1737.49418

**Published:** 2009

**Authors:** AlaEldin H. Ahmed, Tarig E. Yagoub, Faris Muthana

**Affiliations:** 1 *Department of Medicine, University of Khartoum, Khartoum, Sudan. E-mail: drahamed@hotmail.com*; 2 *Elshaab Teaching Hospital, Khartoum, Sudan*; 3 *Sudan Heart Centre, Khartoum, Sudan*

Sir,

Many studies have shown that comorbidity with regard to chronic obstructive pulmonary disease (COPD) and coronary artery disease (CAD) exists at different rates; and that especially, they share a common risk factor, namely, cigarette smoking.[1] COPD and CAD comorbidity affects quality of life and increases mortality.[2] The incidence and prevalence of CAD are increasing in the developing world.[3] This is caused by the rapid socioeconomic growth in developing countries, increasing exposure to risk factors for CAD, such as diabetes, hypercholesterolemia, hypertension and smoking.[3] In the developed world, COPD affects more than 1 in 20 of the adult population; and over the next few years, COPD is projected to be the third leading cause of death.[4] Little is known about the epidemiology of COPD in the developing world; in fact, there is hardly any published work in relation to this. The present study was designed to estimate the prevalence of COPD in patients with catheter-diagnosed CAD, and to describe factors that may increase the likelihood of COPD and CAD comorbidity in Sudan — a developing-world country.

This is a cross-sectional study that included all consecutive adults with catheter-diagnosed CAD recruited from 2 cardiac centers in Khartoum, Sudan — Sudan Heart Centre and Elshaab Teaching Hospital — during a 6-month period starting August 2008. Ethical approval for the study was obtained from the administrative and ethical committee of Sudan Heart Centre, and all patients gave informed consent to take part in the study. All patients were interviewed and basic demographic data was collected. Patients performed spirometry using an electronic spirometer: *Spida 5, Micromedical, England*. The maneuver was explained to each subject, and the best of three readings was recorded. Height was measured to the nearest centimeter, and weight was recorded to the nearest kilogram. Predicted values were calculated as those for Blacks. Study diagnoses were based on guidelines developed by the Global Initiative for Chronic Obstructive Lung Disease.[4] A study diagnosis of COPD was assigned to persons with FEV1 less than 80% predicted.[4]

A total of 59 patients with catheter-diagnosed CAD were studied. Of the 59 patients, 40 performed spirometry; and of these, 11 (28%) had FEV1 less than 80% predicted and were diagnosed as COPD. Table 1 shows the demographic and clinical characteristics of patients with CAD and COPD comorbidity and of those with CAD comorbidity alone. Among patients with CAD and COPD comorbidity, 73% were male; and 64% were either current or previous smokers compared with 38% among patients with CAD alone. Seventy-three percent of patients with COPD and CAD had single-vessel disease, whereas 66% of those who had CAD alone had multiple-vessel disease.

**Table 1:** Demographic and clinical characteristics of patients with spirometry-diagnosed COPD and of those who do not have COPD among patients with CAD

Characteristic	COPD + CAD 11 (28%)	CAD alone 29 (72%)	Total (40)
Age mean ± SD	46 ± 3.0	43 ± 2.4	
Male	8 (73)	17 (59)	25
Female	3 (27)	12 (41)	15
Current or previous smokers	7 (64)	11 (38)	18
Single-vessel disease	8 (73)	10 (34)	18
Multiple-vessel disease	3 (27)	19 (66)	22

The present study has shown that more than 1 in 4 patients with CAD had concomitant COPD. Prior work has demonstrated that CAD and COPD comorbidity exists at different rates.[1–2] The difference between previous work and our study with regard to prevalence may be explained by the fact that the present study was hospital based and had recruited patients with CAD diagnosed by cardiac catheterization. Of importance, however, is the fact that the present study was conducted in a developing-world country, where the epidemiology-related factors of CAD and COPD are likely to be different compared with those in the developed world. Nevertheless, the prevalence rate recorded in this study is high enough to warrant COPD screening of patients with catheter-proven CAD. Epidemiological studies have shown that the developing world will bear the brunt of the worldwide increasing incidence of CAD and consequent mortality.[3] Given these projected increases in CAD, the incidence of COPD comorbidity in CAD patients shown in this study will have an enormous burden in terms of disability, mortality and health expenditure in developing-world countries. This will be an addition to health budgets that are already exhausted by the load of treating and preventing endemic diseases.

Our study has shown that there are more patients with single-vessel disease among those with CAD and COPD comorbidity than among those with CAD alone. The likely explanation for this finding is a selection bias in the population we studied. It is known that concomitant COPD in CAD patients increases symptoms.[1,2] The patients we studied were patients with CAD who were recruited from the catheter laboratory, and it is very likely that they were symptomatic and were, therefore, investigated by cardiac catheterization. However, a larger-scale study is needed to find if concomitant COPD affects prognosis in patients with single-vessel CAD.

The number and percentage of smokers among patients with CAD alone and CAD and COPD comorbidity in the present study fall short of figures recorded in other epidemiological surveys.[4–5] We believe that the likely explanation for these differences is cultural denial of smoking in our community, especially among females. This is supported by epidemiological studies of smoking conducted in communities similar to ours, using questionnaires; these studies showed wide variation in prevalence and concluded that social, cultural and religious inhibitions may have prevented smokers from providing accurate information about their smoking habits.[6] We therefore consider that the actual number of smokers among the population we studied is much higher. Nevertheless, the percentage of smokers among patients with CAD and COPD comorbidity is higher than among patients with CAD comorbidity alone, indicting that smoking may have been a risk factor for CAD and COPD comorbidity. There are, however, 2 further alternative explanations for the low prevalence of smoking among the present study population that we ought to consider. Firstly, we have not assessed the role of passive smoking, which increases the risk of both ischemic heart disease and COPD.[7] Hence we cannot rule out the possible contribution of passive smoking to causing COPD and CAD in our subjects. Secondly, it is possible that nonsmoking-related COPD and risk factors other than smoking for CAD were important etiological factors in this study population.

The 2 limitations of this study are that the numbers we studied were small; and that reduction in lung function may have been caused by diseases other than COPD, such as asthma, or, at least in part, may have resulted from heart failure in some patients. However, the proportion of these patients is unlikely to be large.

In conclusion, this study has shown that in Sudan, a developing country, 28% of patients with catheter-proven CAD have concomitant COPD. Such high incidence will have enormous burden on health expenditure in a region where health budgets are already strained by costs of combatting endemic diseases. Given the projected increases in incidence of CAD in this part of the world, the problem is likely to get worse. The prevalence rate we recorded warrants routine screening of CAD patients for COPD. Further larger-scale studies are needed to determine future trends and define the epidemiology of COPD more precisely.
